# Preterm birth and risk for language delays before school entry: A sibling-control study

**DOI:** 10.1017/S0954579419001536

**Published:** 2021-02

**Authors:** Imac Maria Zambrana, Margarete E. Vollrath, Bo Jacobsson, Verena Sengpiel, Eivind Ystrom

**Affiliations:** 1 Department of Special Needs Education, University of Oslo, Oslo, Norway; 2 The Norwegian Center for Child Behavioral Development, Oslo, Norway; 3 Department of Mental and Physical Health, Norwegian Institute of Public Health, Oslo, Norway; 4 Section of Health, Developmental and Personality Psychology, Department of Psychology, University of Oslo, Oslo, Norway; 5 Department of Obstetrics and Gynaecology, Sahlgrenska Academy, Gothenburg University, Sweden; 6 Department of Genetics and Bioinformatics, Domain of Health Data and Digitalisation, Norwegian Institute of Public Health, Oslo, Norway; 7 Department of Obstetrics and Gynaecology, Sahlgrenska University Hospital, Gothenburg, Sweden; 8 Department of Mental Disorders, Norwegian Institute of Public Health, Oslo, Norway; 9 PharmacoEpidemiology and Drug Safety Research Group, School of Pharmacy, University of Oslo, Oslo, Norway.

**Keywords:** preterm birth, language delay, sibling control study, cohort study, early childhood

## Abstract

We investigated whether children born preterm are at risk for language delay using a sibling-control design in the Norwegian Mother and Child Cohort Study (MoBa), conducted by the Norwegian Institute of Public Health. Participants included 26,769 siblings born between gestational weeks 23 and 42. Language delay was assessed when the children were 1.5, 3, and 5 years old. To adjust for familial risk factors, comparisons were conducted between preterm and full-term siblings. Pregnancy-specific risk factors were controlled for by means of observed variables. Findings showed that preterm children born before week 37 had increased risk for language delays at 1.5 years. At 3 and 5 years, only children born before week 34 had increased risk for language delay. Children born weeks 29–33 and before week 29 had increased risk for language delay at 1.5 years (RR = 4.51, 95% CI [3.45, 5.88]; RR = 10.32, 95% CI [6.7, 15.80]), 3 years (RR = 1.50, 95% CI [1.02, 2.21]; RR = 2.78, 95% CI [1.09, 7.07]), and 5 years (RR = 1.63, 95% CI [1.06, 2.51]; RR = 2.98, 95% CI [0.87, 10.26]), respectively. In conclusion, children born preterm are at risk for language delays, with familial confounders only explaining a moderate share of the association. This suggests a cause-effect relationship between early preterm birth and risk for language delay in preschool children.

Preterm birth is associated with neurodevelopmental risks, such as lower language skills in toddlers and preschoolers (Guarini et al., 2009; Menyuk, Liebergott, & Schultz, 1995; Palumbi et al., 2018; Ribeiro et al., 2011; Wolke, Samara, Bracewell, & Marlow, 2008; Zambrana, Vollrath, Sengpiel, Jacobsson & Ystrom, 2016). This might be explained by the fact that the third trimester is crucial for fetal brain development, with rapid development of neurons and wiring. This association has been found to be inverse linear, i.e., shorter gestational length is related to poorer language skills and higher risks for language delays (Boyle et al., 2012; de Jong, Verhoeven, & van Baar, 2012; Dong, Chen, & Yu, 2012; McGowan, Alderdice, Holmes, & Johnston, 2011; Quigley et al., 2012; Stene-Larsen et al., 2014). On one hand, it is expected that the preterm children's language development will to a certain degree catch up with time (Fitzpatrick, Carter, & Quigley, 2016; Luu, Vohr, Allan, Schneider, & Ment, 2011; Luu et al., 2009). On the other hand, early severe language delays, which are prevalent in children of the lowest gestational age, predict a higher risk for persistent language delays throughout the preschool years. These delays are in turn related to academic struggles as well as socioemotional and behavioral problems (Beitchman et al., 2001; de Jong et al., 2012; Dong et al., 2012; Wolke et al., 2008). Therefore, one major question is whether and for how long the early risks for language delays persist.

A meta-analysis that compiled longitudinal change and stability trends from several smaller studies found that preterm-born children still scored lower than term-born children on selected language tests at age 12 years (van Noort-van der Spek, Franken, & Weisglas-Kuperus, 2012). Most earlier studies have adjusted for an array of well-known risk factors for preterm birth, among them family risk factors such as socioeconomic status, and maternal risk factors, such as hypertension or smoking, which may be confounded with language delays (Hauge, Aarø, Torgersen, & Vollrath, 2013). However, the associations between gestational age and language delays may also be caused by unmeasured family risk factors such as a common genetic risk or a genetic risk for language delay that is related to risk-related maternal behavior that affects the prenatal environment and risk for preterm birth (Bishop, Price, Dale, & Robert, 2003; Kovas et al., 2005). For example, previous studies have found that not only prenatal and neonatal health complications predict negative outcomes in preterm-born children (Hauge et al., 2013; Poehlmann-Tynan et al., 2015) but also negative parenting style (Poehlmann-Tynan et al., 2015), which can be considered to be relatively stable across siblings and strongly related with genetic contributions to language skills (Dale, Tosto, Hayiou-Thomas, & Plomin, 2015). Traditional case–control and cohort designs do not allow taking such factors into account in a satisfactory way. This raises the question of the extent to which language delay is distinctively associated with gestational age as compared with unmeasured family factors.

Sibling-control studies provide a stronger design by comparing language skills among siblings who are discordant with regard to gestational age but share both genes and family background (Brandlistuen, Ystrom, Nulman, Koren, & Nordeng, 2013; D'Onofrio et al., 2013; Susser, Eide, & Begg, 2010; Zambrana et al., 2016). In addition, sibling studies allow for the control of observed risk factors that may vary between pregnancies in the same family (e.g., hypertension or maternal smoking; Hauge et al., 2013). Such adjusted sibling designs consequently distinguish between effects that are due to family-invariant (e.g., stable) factors, pregnancy-specific factors, and gestational age (Zambrana et al., 2016). Using a sibling-control design, we recently presented findings showing that risk for language delays in children that were born preterm decreased between 1.5 and 3 years. We also showed (for spontaneous births) that any remaining influence of preterm birth was explained by familial and pregnancy-related risk factors (Zambrana et al., 2016).

The objective of this study was to extend the follow-up period of roughly the same sample to 5 years in order to understand whether the association between gestational age and risk for language delays continues to decline as children approach school age and to identify factors that may explain any remaining association. More specifically, we used a sibling-control design to examine the nature of the associations between (a) gestational age at birth and relative risks for language delays from age 1.5 to 5 years, and (b) whether the associations are brought about by stable (e.g., genetic risk) and variable (e.g., perinatal conditions) risk factors within families or the direct effects of gestational age at birth. The current study builds on and extends previous work by examining the risks for language delays that are associated with preterm birth at a period when children are approaching school age, an age when language delays have been found to be associated with general learning challenges with potential effects on children's well being.

## Method

### Sample

This study is based on data from the Norwegian Mother and Child Birth Cohort Study (MoBa), which is conducted at the Norwegian Institute of Public Health (Magnus et al., 2006). The sample for MoBa includes 114,744 children and 95,242 mothers, who were recruited from across Norway during the period 1999–2008 in connection with an ultrasound examination that is routinely offered around pregnancy week 18. Informed consents were obtained upon recruitment, and 40.6% consented to participate. With respect to procedures, MoBa has received a license from the Norwegian Data Inspectorate and approval from The Regional Committee for Medical Research Ethics. The current study used data from the Medical Birth Registry of Norway (MBRN) and maternal-reported MoBa questionnaires at the child chronological ages of 1.5 years, 3 years, and 5 years (the response rates were 77, 62, and 50% of those giving informed consent, respectively), and it is based on version 8B of the quality-assured data files that were released in 2015 (http://www.fhi.no/moba-en). Starting with 33,026 children of mothers that were participating in MoBa with several pregnancies, only first-borns from multiple births with either older or younger participating siblings from other pregnancies were included. We only retained children having outcome data at 1.5 years (*n* = 24,665), 3 years (*n* = 19,795), or 5 years (*n* = 13,394), amounting to a total of 26,769 (51.5% boys) children nested within 14,703 maternal clusters (see Table 1 for descriptive sample information). Of the 26,769 children, 2,104, 6,974, and 13,375 had missing data at 1.5, 3, and 5 years, respectively. In addition to nonresponse, the high amount of missing data at 5 years was because the questionnaire was not sent out to the cohorts of children who had already turned 6 years.

**Table 1.** Descriptive characteristics of the sibling sample from MoBa (*N* = 26,769)

Variables	*N* (*M*±*SD*)	*N* Missing
*Child*		
Gestational age at birth, weeks	(40.0±1.7)	0
Boys	13,784	0
Malformations at birth	1,262	0
SGA	398	17
*Pregnancy*		
Parity (more than one pregnancy)	15,527	0
Twin birth	242	0
Hypertensive disease	1,388	0
Bleeding between weeks 13–28	418	0
Recurrent urinary tract infections	796	0
Gestational diabetes	343	0
Nonspontaneous delivery	4,770	0
*Mother*		
BMI > 30.0 kg/m^2^	2,257	832
Depressive and anxiety symptoms	1,359	518
Smoking during pregnancy	1,641	49
Alcohol during pregnancy	7,678	952
Age mother, years	(29.9±4.1)	0

*Note*: *N*, sample size; M, mean; *SD*, standard deviation; SGA, small for gestational age; BMI, body mass index.

### Preterm birth

Gestational age at birth was determined by ultrasound examinations or on the date of last menstruation registered in MBRN (for 2% of the sample). Five gestational age groups were created: *very early preterm* (gestation weeks ≤ 27/6), *early preterm* (weeks 28/0 to 33/6), *late preterm* (weeks 34/0 to 36/6), *early term* (weeks 37/0 to 38/6), and *full term* (≥ weeks 39/0). We calculated the average age of gestation across all children of each mother (i.e., group mean). In the sibling-control analyses, the differences between the average gestational age of the sibling group and the individual score provided gestational age deviations from the particular family mean (Zambrana et al., 2016)

### Language delay risk

Risks for *language delays* at ages 1.5, 3, and 5 years were measured by maternal-reported items from the Ages and Stages Questionnaires (ASQ) communication subscales (Dale et al., 2015), for which good test-retest agreement and concurrent validity have been reported (Janson & Squires, 2004; Richter & Janson, 2007; Squires, Bricker, & Potter, 1997). For 1.5-year-olds, MoBa selected four of the six age-corresponding ASQ items. For 3-year olds, MoBa selected four of the six age-corresponding ASQ items and mixed this with two items from the ASQ scales for ages 1.5 and 4 years. For 5-year-olds, MoBa included all six original items from the 5-year Communication scale plus one item from the original 4-year scale. The response categories were 1 = *yes*, *very often*, 2 = *yes*, *sometimes*, and 3 = *not yet*. In order to take into consideration that each item discriminates children at different loci on the latent continuum of risk for language delay in the child population, we examined the item function and reliability at different loci of the scales by using item response theory analyses. To get a more reliable basis for making cut points, we subsequently saved out factor scores separately for the three points. Following Zambrana et al. (2014), the cut points for risk of language delay were set at 1.5 standard deviations (*SD*) above the mean at all ages (Squires et al., 1997).

### Confounders

We controlled for risk factors related to preterm birth that can be unique to the specific pregnancy as well as for the health status of the mother and child in the given pregnancy. Pregnancy-related confounders were parity, multiple birth status, hypertensive condition, bleeding between weeks 13 and 28, recurrent urinary tract infections, gestational diabetes, and type of onset of delivery (i.e., nonspontaneous or spontaneous). Mother-related confounders were high body mass index (>30 kg/m^2^), levels of self-reported anxiety and depressive symptoms above the clinical cutoff on the Symptom Checklist-5 during pregnancy (Strand, Dalgard, Tambs, & Rognerud, 2003), smoking and alcohol intake during pregnancy, and maternal age. Child-related confounders were sex, serious malformations at birth, and being small for gestational age (> 2 *SD* below the uterine growth curve; Maršál et al., 1996).

### Statistical Analyses

All of the analyses were run in STATA 15.0 statistical software, with all three models (i.e., unadjusted cohort, unadjusted sibling, and adjusted sibling-control analyses) using the same sibling sample for comparison purposes (StataCorp, 2017). First, we compared the preterm groups and the full-term group by applying a regular cohort design. Subsequently, we planned to adjust our crude associations in two steps: first, adjusting for all risk factors common to siblings by adjusting for the mother's average time of delivery (sibling control) and second, adjusting for risk factors specific to each pregnancy by using observed covariates (adjusted sibling control) For details about the sibling models see (Zambrana et al., 2016). We modeled risk for language delay across time by using a Poisson mixed model with a random intercept (set at age 5 years) and a fixed slope for effects at 1.5 and 3 years. Importantly, we subtracted gestational age from (and added time of reporting to) the panel data time variable set to calendar birth day, effectively centering each child´s measurement to its biological age as given by date of gestation and date of report. In this way, the effect of preterm birth could not be explained by different timing of measurement. To model the effect of age of gestation we allowed for a fixed quadratic effect of time. In the sample, we had four sibling cluster groups with varying numbers of children in them: Cluster 1 (children with no siblings), *n* = 3,316; Cluster 2 (children with 1 sibling), *n* = 21,454; Cluster 3 (children with 2 siblings), *n* = 1,923; and Cluster 4 (children with 3 siblings), *n* = 76. The overall, between-mother, and within-mother standard deviation of gestational age was 12, 10, and 7 days. We calculated the relative risk point estimates at the selected weeks of gestation for the three different outcome points by using the linear combination of estimates (lincom) function of STATA (https://www.stata.com/manuals/rlincom.pdf).

As with most cohort studies, the voluntary participation and attrition is far from random in MoBa (R. Nilsen et al., 2009; R. M. Nilsen et al., 2013). We have recently shown that the earliest preterm births are slightly underrepresented in MoBa, while the older preterm births are somewhat overrepresented (Zambrana et al., 2016). We estimated missing data in the outcome variables by using full information maximum likelihood and all of the available outcome data so that the full analysis sample was 57,854 measures of risk for language delay, nested within 26,769 children, nested within 14,703 mothers.

## Results

The unadjusted cohort results in Table 2 (left column) show that early preterm birth is associated with increased risk for language delay at all ages and that this risk is inversely linear within each point. For instance, the very early preterm group had an elevenfold increase in relative risk (RR) at 1.5 years compared with the reference term group, RR = 11.49, 95% CI [8.55, 15.49], while the RRs for the early preterm, the late preterm, and the early term groups were correspondingly 5.37, 95% CI [4.46, 6.48]; 2.56, 95% CI [2.29, 2.87]; and 1.81, 95% CI [1.65, 1.99], respectively, at this age. By age 5 years, the very early preterm group still showed a threefold increase in RR for language delay compared with the reference term group, RR = 3.19, 95% CI [0.96, 10.57], while the early term group only showed a 1.06 increased risk at this age, 95% CI [0.98, 1.16].

**Table 2.** Relative risk (RR) for language delay at 1.5, 3, and 5 years by gestational weeks (*N* = 26,769)

	Unadjusted cohort^a^			Unadjusted sibling control^b^			Adjusted sibling control^c^		
Term and preterm groups^d^	RR	95% CI		RR	95% CI		RR	95% CI	
1.5 years									
Full term	1.00	−	−	1.00	−	−	1.00	−	−
Early term	1.81	1.65	1.99	1.83	1.64	2.03	1.82	1.64	2.03
Late preterm	2.56	2.29	2.87	2.60	2.24	3.01	2.42	2.09	2.80
Early preterm	5.37	4.46	6.48	5.53	4.27	7.16	4.51	3.45	5.88
Very early preterm	11.49	8.55	15.45	11.20	7.26	17.28	10.32	6.74	15.80
3 years									
Full term	1.00	−	−	1.00	−	−	1.00	−	−
Early term	1.97	1.62	2.41	0.83	0.73	0.94	0.83	0.73	0.94
Late preterm	2.49	1.89	3.28	1.06	0.86	1.29	0.99	0.81	1.21
Early preterm	4.19	2.69	6.56	1.81	1.24	2.65	1.50	1.02	2.21
Very early preterm	7.30	2.89	18.42	2.95	1.14	7.65	2.78	1.09	7.07
5 years									
Full term	1.00	−	−	1.00	−	−	1.00	−	−
Early term	1.06	0.98	1.16	1.04	0.95	1.15	1.04	0.94	1.15
Late preterm	1.29	1.06	1.58	1.27	1.02	1.59	1.18	0.95	1.47
Early preterm	2.02	1.37	2.97	2.01	1.32	3.08	1.63	1.06	2.51
Very early preterm	3.19	0.96	10.57	3.04	0.82	11.30	2.98	0.87	10.26

*Note*: ^a^Analyses with the same sibling sample used in a conventional cohort design. ^b^Analyses using the sibling-control design, controlling for unobserved familial risks, but not for measured confounders. ^c^Analyses using the sibling-control design, controlling for unobserved familial risks, and for child sex, multiple birth status, serious malformations at birth, small for gestational age, parity, pregnancy smoking and alcohol intake, hypertensive condition, bleeding between weeks 13 and 28, recurrent urinary tract infections, gestational diabetes, and nonspontaneous delivery as well as high maternal BMI, levels of self-reported anxiety and depressive symptoms during pregnancy, and maternal age. ^d^Gestational weeks and groups: ≥ 39/0 weeks = Full term, 37/0–38/6 weeks = Early term, 34/0–36/6 weeks = Late preterm, 28/0–33/6 wks = Early preterm, and ≤ 27/6 weeks = Very early preterm.

Adjusting for family-invariant risk factors (sibling control) did not alter the estimates considerably at 1.5 years (see Table 2, middle column). However, family-invariant factors accounted for a substantial amount of the sizable associations found at 3 years. At this period, only the very early preterm and early preterm groups showed an increased risk for language delay, which now dropped from a sevenfold to a threefold increased risk and a fourfold to less than a twofold increased risk, respectively. At 5 years, although the associations that were found in the cohort analyses were not accounted for by family-invariant factors, the initial associations were less sizable in the cohort analysis at this age.

Largely, adjusting for pregnancy-specific factors (observed covariates) did not alter the estimates considerably after the sibling control (Table 2, right column). Neither invariant nor variant familial factors accounted for much of the risk at 1.5 years. All preterm gestational age groups were still associated with an increased risk for language delay at 1.5 years, with associations increasing linearly from RR = 1.82, 95% CI [1.64, 2.02], for children born early term to RR = 10.32, 95%CI [6.74, 15.80], for children born very preterm. At 3 years, only the early preterm, RR = 1.50, 95% CI [1.02, 2.21], and very preterm, RR = 2.78, 95% CI [1.09, 7.07], siblings had a higher risk for language delay than their full-term siblings did. At age 5 years, siblings that were born early and very early preterm had an elevated risk for language delay, RR = 1.63, 95% CI [1.0, 2.51] and RR = 2.98, 95% CI [0.87, 10.26], respectively. However, the confidence intervals indicate substantial uncertainty in the exact effect sizes.

Previous work from the same cohort found that risks for language delays at 18 and 36 months were higher for provider-initiated births than for spontaneous births following sibling control (Zambrana et al., 2016). Thus, to assess the potential differential effects of preterm birth on risk for language delay across spontaneous and provider-initiated births, we compared the three models with and without this interaction. There were no differential effects of preterm birth on risk for language delay according to provider-initiated vs. spontaneous birth for the three models: unadjusted cohort, χ^2^ (7) = 1.58, *p* = .98; *AIC* = -12.42, unadjusted sibling control, χ^2^ (8) = 6.20, *p* = .62; *AIC* = -9.80, adjusted sibling control, χ^2^ (7) = 3.27, *p*=.86; *AIC* = -10.73. However, neither the current growth models nor the measures are the same as in Zambrana et al. (2016).

## Discussion

Overall, we found that lower gestational age was associated with a linear increase in risk of language delay at all ages. However, the finding that there is a diminished risk for language delays over time and that most preterm children are not born at very early gestational ages indicates that the majority of the preterm children have caught up with their siblings by age 5. When examining the degree to which other factors could account for the increased risks, the results varied according to age. At age 1.5 years, the sibling-control analyses showed that the effects of preterm birth on risk for language delays were substantial and not due to factors that are stable within the families or covariates specific to the pregnancies. At age 3 years, however, family invariant and pregnancy-specific confounders accounted for persistent relative risk for language delays to a large degree, except for the very early preterm group. At 5 years, a similar pattern was evident, but the effects in the unadjusted analyses were much smaller initially.

The results are both in line with and in contrast to findings from previous studies. First of all, prior studies have also found that preterm birth is a major risk factor for early language delays (Menyuk et al., 1995; Ribeiro et al., 2011; Zambrana et al., 2016) and that the delays are less pronounced by age 3 years (Zambrana et al., 2016). This study adds to the literature by showing that, although the unadjusted analyses show that the risk for language delays is decreasing further throughout the preschool years, the language delays appear to be more stable in this period following the sibling adjustment. In fact, the earliest preterm born children are in particular continuing to display a considerable increased risk for language delay at 5 years. Future studies will show to what extent this risk decreases further as the children enter school.

Secondly, the sibling-control models enabled us not only to examine the role of the measured covariates but also to adjust for the effects of unmeasured familial risk factors. In fact, our sibling design revealed that family factors accounted for a substantial amount of the increased and continued risk of language delay that the preterm-born children showed up to ages 3 and 5 years. This is in line with our previous findings about the association between preterm birth and language production and comprehension up to 3 years, at least for children that were delivered spontaneously (Zambrana et al., 2016; Ask et al., 2018) recently found preterm birth to be associated with innatention, but not hyperactivity, within sibling pairs. Therefore, language delay and inattention could be markers of a broader spectrum of brain health risk following preterm birth.

Some limitations should be mentioned. The ASQ communication subscales are short scales that were developed for screening purposes, especially at 18 months, implying that the reliability is strongest at the low pole of the scales. Therefore, while our findings address the degree to which gestational age is associated with a decrease in relative risk for early language delays (i.e., a catch-up), more research is needed to examine subtler differences at more advanced levels of language. Yet, the growth-model design ensures that all associations are estimated simultaneously and allows for adjusting the time of measurement for age of gestation and time of maternal report, so it provides more robust measures than does examining one point at the time. It should also be mentioned that the sibling-control design reduces the statistical power of the analyses. In particular, the 5-year estimates for the very early preterm birth group have large and uncertain confidence intervals. For example, while there was a significant association between early preterm birth and language delay at 5 years, the confidence intervals for very early preterm crossed 1.00. However, the most likely value (i.e., the point estimate) for very early preterm birth was 2.98 not 1.00. It would be illogical to argue based on the conventions of statistical significance that it is “unsafe” to be born early preterm and at the same time “safe” to be born very early preterm.

Finally, given that mothers with preterm birth are more likely to drop out of the study (introducing bias), as presented in our previous paper (Zambrana et al., 2016), and that this risk is increased further if the children also have language delays, there may be an additional bias in the rates of language delays in our sample such that they are also more prone to the effects of mothers who have dropped out of the study. Including all of the available outcome data and estimating missing data by using full information maximum likelihood addresses some of the problems with attrition. However, sibling-design estimates can also be biased if the covariates that are included are more familial than the exposure and random measurement error is not accounted for (Frisell, Öberg, Kuja-Halkola, & Sjölander, 2012).

Altogether, the present and previous findings suggest that children that are born late preterm to early term catch up with their peers before starting school. However, even if the risk for language delays diminishes with age, the delays are still substantial for children born before week 34, especially for children born very early preterm, who still display a striking threefold relative risk at both ages 3 and 5. Considering the importance of language abilities for cognitive and social adjustment as well as mental health (Beitchman et al., 2001; de Jong et al., 2012; Dong et al., 2012; Wolke et al., 2008), all of which are crucial in transition to school, this is of particular importance to practitioners working with this group of children. Of particular practical significance, the results of the present study also suggest that the etiological picture for children who are born preterm who display persistent risk for language delays may be more complex than that of children who catch up with their peers. However, additional studies are needed to further our understanding of the complexity of the delays and the factors underlying them.

## Conclusion

This population-based sibling-cohort study shows that preterm birth is associated with an increased risk for language delays at 1.5, 3, and 5 years of age. Moreover, this association is not confounded by familial risk factors and it is potentially causal in nature. However, the magnitude of this risk decreases as the children approach school age. This suggests that the factors explaining the increased risk for language delay in children who are delivered preterm change as these children approach school age, which potentially can have implications for practitioners who are working with preterm-born children with persistent risk for language delays.

## Supplementary material

For supplementary material accompanying this paper visit http://dx.doi.org/10.1017/S0954579419001536. click here to view supplementary material
