# Supplementary material for: Preterm birth and risk for language delays before school entry: A sibling-control study
Source: Dev Psychopathol. 2021 Feb;33(1):47–52. doi: 10.1017/S0954579419001536 (PMC7900651; doi:10.1017/S0954579419001536)
Supplement: Supplementary file 1 [file S0954579419001536sup001.docx]

**Supplementary Table with RR estimated for 6 categories of gestational weeks**

**Table 2.** Relative risk (RR) for language delay at 1.5, 3 and 5 years by gestational weeks (N = 26,769)

|  |  | Unadjusted cohorta | | |  | Unadjusted sibling controlb | | |  | Adjusted sibling controlc | | |
| --- | --- | --- | --- | --- | --- | --- | --- | --- | --- | --- | --- | --- |
| Term and preterm groupsd | | RR | 95% CI | |  | RR | 95% CI | |  | RR | 95% CI | |
| 1.5 years | |  |  |  |  |  |  |  |  |  |  |  |
|  | Full term | 1.00 | - | - |  | 1.00 | - | - |  | 1.00 | - | - |
|  | Early term | 1.81 | 1.65 | 1.99 |  | 1.83 | 1.64 | 2.03 |  | 1.82 | 1.64 | 2.03 |
|  | Late preterm | 2.56 | 2.29 | 2.87 |  | 2.60 | 2.24 | 3.01 |  | 2.42 | 2.09 | 2.80 |
|  | Moderate preterm | 4.93 | 4.12 | 5.89 |  | 5.07 | 3.97 | 6.48 |  | 4.18 | 3.25 | 5.39 |
|  | Very preterm | 8.05 | 6.52 | 9.95 |  | 8.35 | 6.15 | 11.33 |  | 6.49 | 4.72 | 8.94 |
|  | Extremely preterm | 11.49 | 8.55 | 15.45 |  | 11.20 | 7.26 | 17.28 |  | 10.32 | 6.74 | 15.80 |
| 3 years | |  |  |  |  |  |  |  |  |  |  |  |
|  | Full term | 1.00 | - | - |  | 1.00 | - | - |  | 1.00 | - | - |
|  | Early term | 1.97 | 1.62 | 2.41 |  | 0.83 | 0.73 | 0.94 |  | 0.83 | 0.73 | 0.94 |
|  | Late preterm | 2.49 | 1.89 | 3.28 |  | 1.06 | 0.86 | 1.29 |  | 0.99 | 0.81 | 1.21 |
|  | Moderate preterm | 1.64 | 1.17 | 2.30 |  | 1.70 | 1.18 | 2.45 |  | 1.43 | 0.99 | 2.06 |
|  | Very preterm | 2.37 | 1.54 | 3.64 |  | 2.47 | 1.57 | 3.90 |  | 1.97 | 1.23 | 3.15 |
|  | Extremely preterm | 7.30 | 2.89 | 18.42 |  | 2.95 | 1.14 | 7.65 |  | 2.78 | 1.09 | 7.07 |
| 5 years | |  |  |  |  |  |  |  |  |  |  |  |
|  | Full term | 1.00 | - | - |  | 1.00 | - | - |  | 1.00 | - | - |
|  | Early term | 1.06 | 0.98 | 1.16 |  | 1.04 | 0.95 | 1.15 |  | 1.04 | 0.94 | 1.15 |
|  | Late preterm | 1.29 | 1.06 | 1.58 |  | 1.27 | 1.02 | 1.59 |  | 1.18 | 0.95 | 1.47 |
|  | Moderate preterm | 1.92 | 1.32 | 2.77 |  | 1.91 | 1.27 | 2.87 |  | 1.57 | 1.04 | 2.37 |
|  | Very preterm | 2.63 | 1.64 | 4.22 |  | 2.66 | 1.59 | 4.45 |  | 2.08 | 1.23 | 3.53 |
|  | Extremely preterm | 3.19 | 0.96 | 10.57 |  | 3.04 | 0.82 | 11.30 |  | 2.98 | 0.87 | 10.26 |

*Note*: aAnalyses with the same sibling sample used in a conventional cohort design. bAnalyses using the sibling-control design, controlling for unobserved familial risks, but not for measured confounders. cAnalyses using the sibling-control design, controlling for unobserved familial risks, and for child sex, multiple birth status, serious malformations at birth, small for gestational age, parity, pregnancy smoking and alcohol intake, hypertensive condition, bleeding between weeks 13 and 28, recurrent urinary tract infections, gestational diabetes, non-spontaneous delivery, as well as high maternal BMI, levels of self-reported anxiety and depressive symptoms during pregnancy, and maternal age. dGestational weeks and groups: ≥ 39/0 wks = Full term, 37/0–38/6 wks = Early term, 34/0–36/6 wks = Late preterm, 32/0–33/6 = Moderate preterm, 28/0–31/6 wks = Very preterm, and ≤ 27/6 wks = Extremely preterm.
